# The Ruptured Pericardial Cyst to the Right Atrium: A Case Report

**Published:** 2020

**Authors:** Freidoun SABZI, Reza FARAJI

**Affiliations:** 1. Preventive Cardiovascular Research Center, Kermanshah University of Medical Sciences, Kermanshah, Iran; 2. Department of Biomedical Engineering, Zagros Higher Education Institute, Kermanshah, Iran

**Keywords:** Pericardial cyst (PC), Pericardial effusion, Mediastinal cyst

## Abstract

The pericardial cysts (PC) are rare congenital anomaly. They are usually asymptomatic or incidentally found during surgery or by an imaging modality. We report a 35-yr-old man referred to Imam Ali Hospital, Kermanshah, western Iran in 2017, with palpitation, chest pain and dyspnea and physical exam revealed sign and symptoms of right atrial compression and tamponade.

## Introduction

Pericardial cysts (PC) as a rare congenital disorder, comprising approximately 7% of all mediastinal mass. The incidence of this lesion between the pericardial masses is estimated to be from 10% to 17%. These cystic masses as the most common benign neoplasm of the pericardium and may have an embryologic base for their occurrences (1,2). They are often detected in the third to fifth decade of life but small cyst may be found even in the 8 ^th^ decade of life and their incidence is equal between both genders. Clinical sign and symptoms of these cystic masses are similar to another neoplasm of the pericardium such as hydatid cyst, loculated pericardial effusion. Thymoma, thymolipoma, lipoma and rare malignant tumors (3,4).

## Case Report

**Fig. 1:**
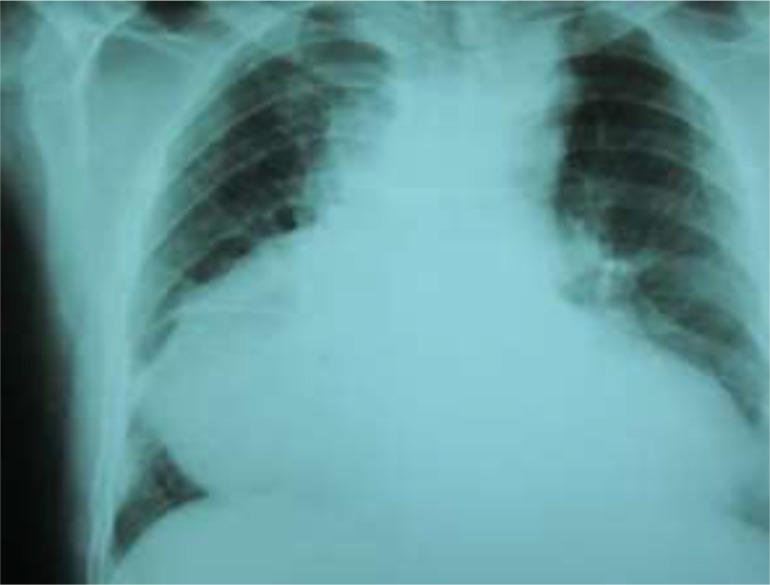
Global cardiac enlargement with dumping of right cardio-phernic angle

A 35-year-old man admitted to our emergency center (Imam Ali Hospital, Kermanshah, western Iran in 2017) with complaint of palpitation, dyspnea and nonproductive cough for recent three weeks. His past medical history except for temporary paroxysmal palpitation was unremarkable. Chest x-ray revealed a dumping in the lower right cardiac border (Fig. 1). Routine laboratory exam including, complete blood count, BUN, creatinine and, NA, K were unremarkable but hepatic function tests were abnormally high. Sedimentation rate and C-reactive protein were in abnormal high ranges. Arterial blood saturation in room air was normal. The routine coagulation profile was normal. Cardiac enzymes were in the normal range.

The patient was not feverish, the pulse was 112 beats/min, and the respiration rate was 22/min. The blood pressure was 110/65 mm Hg. He was tachypneic and tachycardic and had low blood pressure (80/50 mm gH). The physical exam also showed the signs of right atrial tamponade with rapid, weak pulse with paradoxical nature, jugular veins were distended and accentuated with inspiration. The physical exam showed, moderate hepatomegaly (3 cm below of costal ridge), pitting lower extremities edema, and vague heart sounds. The lungs sound was clear, and the neurologic examination was unremarkable. Electrocardiography showed normal sinus rhythm at a rate of 118 beats/min with normal intervals with no ST segments change in the cardiac leads. The electrocardiogram showed low volt QRS height, with T-wave flattening in all of the ECG leads. Due to lung air interposition, a transthoracic echocardiogram was unremarkable without obtaining a diagnostic benefit, and a transesophageal two-dimensional echocardiogram was denied by the patient.

**Fig. 2:**
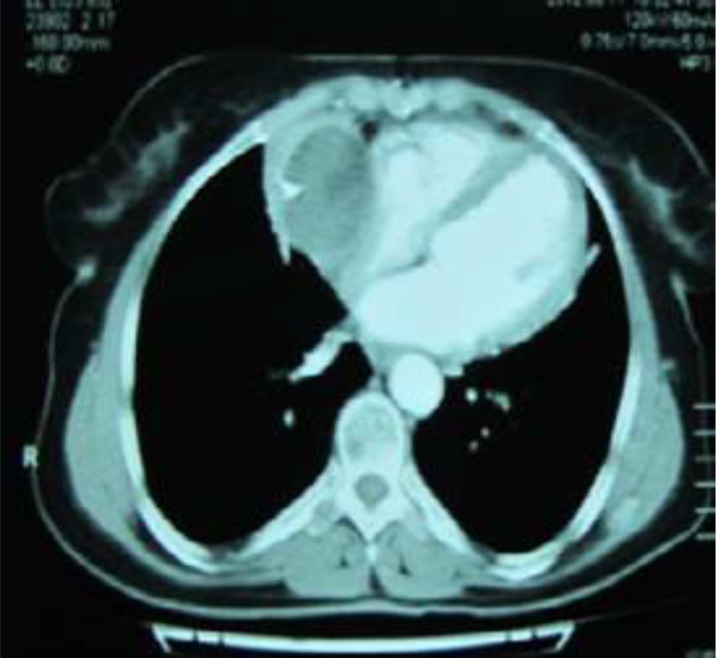
Intra pericardial cyst attached to right atrium

The cystic lesion closely attached to the surrounding cardiac structures, most probably representing a hydatid cyst (Fig. 1). CT scan was added to imaging modality that showed a large para cardiac cystic mass closely related to cardiac structures (Fig. 2).

**Fig. 3:**
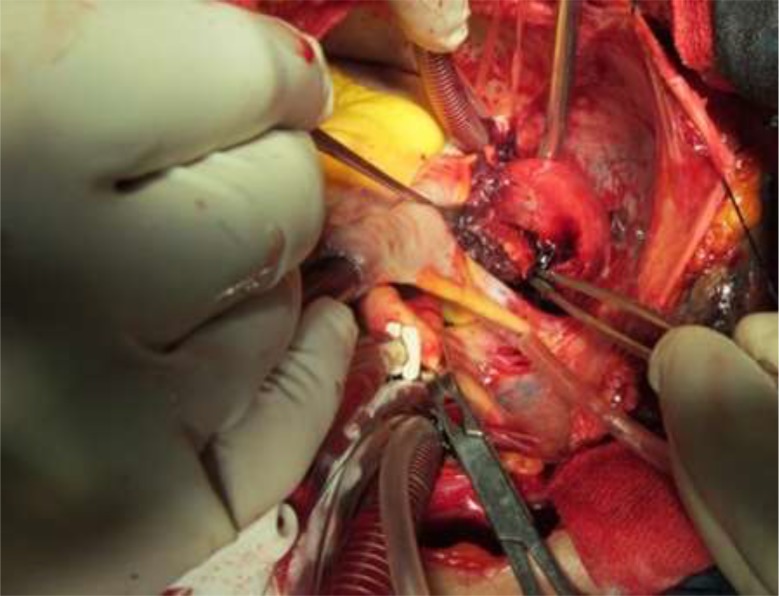
Intra operative view of pericardial cyst

**Fig. 4:**
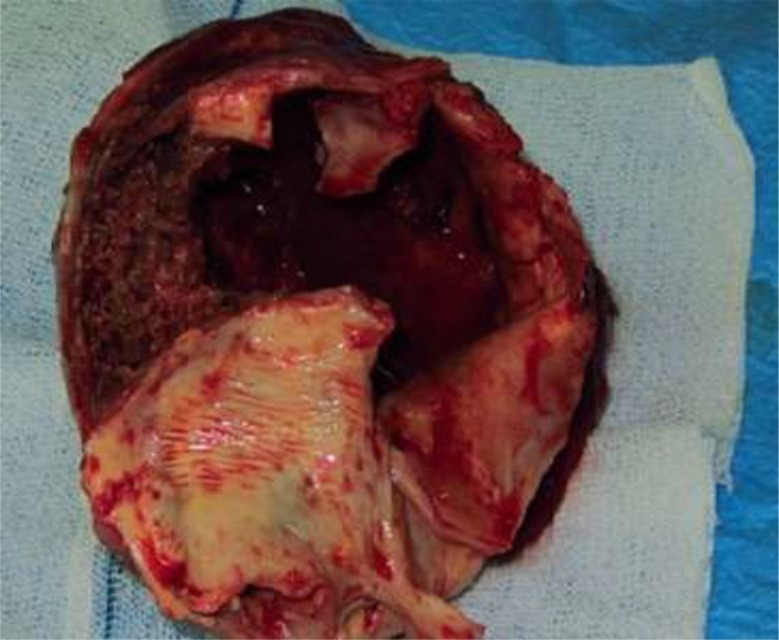
Resected cyst with thick and inflamed wall

With the impression of ruptured hydatid cyst, the patient scheduled for open surgery performed by midline sternotomy and with beating off-pump approach. During intraoperative inspection, he had an intrapericardial cystic mass compressing the right atrium and inferior border of the right hemi-diaphragm. There was mild sero-sanguineous pericardial effusion (Fig. 3). The cystic mass had a thick wall with severe inflammatory reactions. Its cavity contained necrotic, fibrinous, and thrombotic materials with some pasty like materials without the presence of the daughter cyst or typical germinal layer as seen in the HC (Fig. 4). The medial wall of cystic mass was attached densely to the right atrium and compressed its cavity. The cyst cavity was filled by thrombotic material caused by presence of small (3 mm) defect in the medial wall of cyst. The venous blood from right atrium leaked to cyst cavity. For prevention of cardiac injury, medial wall of cyst left open to pericardial cavity, but others part as lateral borders adhered to partial pericardium was easily released and excised.

**Fig. 5:**
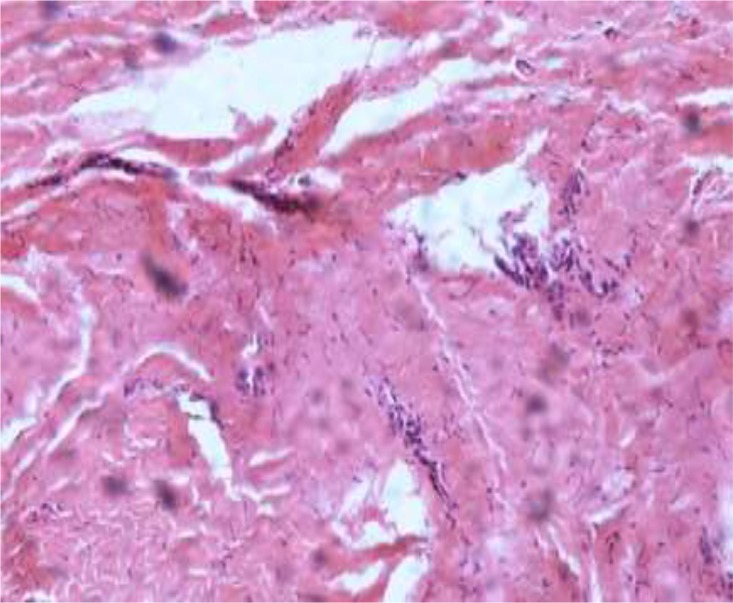
The cyst wall was composed of collagen, with scattered elastic fibers, and was lined by mesothelial cells with foci of hyperplastic mesothelial cells (H,E 100)

Erosion of cyst wall to thin atrial wall caused silent bleeding to cyst cavity and subsequent thrombus formation. No connecting vascular pedicle from cyst to aorta or great vessels was found. On histopathological exam, some evidence of necrotic, fibrinous and thrombotic material with an inflamed pericardial layer were found and no malignant tissue or cells were detected (Fig. 5).

The postoperative course was uneventful and discharged in 6 ^th^ day of operation and in the 6 ^th^ months of follow-up, no recurrence of effusion was detected by TEE as his clinical condition was good.

## Discussion

Pericardial cysts occur because of an aberrant embryologic process in which from mesenchymal diverticula that form the future pericardial sac a bud-like outgrowth protrudes to the lateral side, separated from main diverticula, and formed a pericardial cyst. Others believed that failure of fusion of two bud leads to the formation of an accessory PC (3).

Near 80% of PC are asymptomatic and usually detected incidentally during a routine evaluation for another’s pathology. Seventy percent of pc is detected at the right border of heart toward the costophrenic angle, and 20% located on the left cardiac border and the remaining ten percent of cyst are found in the posterior or anterior mediastinum (4). It needs to verify if the size or location of the PC correlates to higher rates of sequels. However most of the pc are asymptomatic but compressive effects of the cyst on the surrounding structures such as diaphragm, cardiac and lung may lead to dyspnea, hiccups, arrhythmia, chest pain, and some cases present with sinus tachycardia, protracted cough, and episodes of pneumonia and bronchitis and respiratory tract infection have been reported (5).

Other complications include inflammation of cyst’s wall; infection of cyst is content and rupture of cyst into surrounding structures resulting from partial erosion of necrotic cyst wall. Rupture commonly occurs in plural cavity however rare cases of pericardial erosion or rupture into pulmonary tissue or main bronchus has been reported and in two separate studies two cases of supra valvar pulmonary stenosis, and compression of right ventricular outflow tract by cyst, have been observed (6).

The spontaneous resolution of a cyst is rare event and, most likely result from erosion of cyst wall and subsequent drainage of cyst contents into the pleural space. The spontaneous resolution of cyst occurs only in non-inflamed and non-infected cases. CT scan with contrast is the imaging modality of choice for proper diagnosis of PC. However, no studies have been confirmed the superiority of one imaging modality on others such as MRI or echocardiography. On contrast CT scan, in the infected PC cases, or with inflammation of the wall the PC, appears as thick-walled with sharp border and homogeneous content. Failure to enhancement is due to intactness of wall in relation to cardiac chambers (7,8).

The three modalities of treatment of PC include following up with symptomatic management, non-surgical intervention by percutaneous cyst content drainage, and unblocked resection of mass. The indications of surgery include huge size, protracted symptoms, unknown nature and behaviour of cyst and prevention of serious complications such as rupture into pericardial cavity tamponade, suffocation by obstruction of respiratory tract and respiratory failure (9, 10).

## Conclusion

The PC are rarely the cause of dyspnea and this symptom may be a cause of patient’s referral to heart centers for further evaluation. Simple lain chest x-ray highlights the presence of a pc verified by further imaging especially with CT scan. Definitive treatment for symptomatic pc is surgical removal.
